# Clinical and Imaging Features of a Congenital Midline Cervical Cleft in a Neonate: A Rare Anomaly

**DOI:** 10.1155/2015/439596

**Published:** 2015-05-21

**Authors:** Rachelle Goldfisher, Pritish Bawa, Zachary Ibrahim, John Amodio

**Affiliations:** ^1^Department of Radiology, SUNY Downstate Medical Center, 450 Clarkson Avenue, Brooklyn, NY 11203, USA; ^2^Department of Pediatrics, SUNY Downstate Medical Center, 450 Clarkson Avenue, Brooklyn, NY 11203, USA

## Abstract

Congenital midline cervical cleft (CMCC) is a rare congenital anomaly. CMCC and its complications and treatment have been well described in ENT, dermatology, and pediatric surgery literature. However, to our knowledge, the imaging work-up has not been reported in the literature thus far. We present a case of CMCC in a neonate with description of clinical presentation and imaging features.

## 1. Introduction

Congenital midline cervical cleft was first described in 1848 by Luschka [1]; however, it was not fully described until Ombredanne in 1946 [2] explained in his textbook of pediatric surgery. CMCC is uncommon and its diagnosis is usually made on clinical examination. Occasionally, however, CMCC may be confused with a thyroglossal duct or branchial anomaly. The treatment of congenital midline cervical cleft is surgery. To avoid complications of longstanding congenital midline clefts such as limitation of extension of neck or impairment of mandibular growth, early intervention is recommended [3].

Imaging may be requested to differentiate CMCC from these lesions or to determine any coexisting lesions [4]. Magnetic resonance imaging (MRI) is the best modality to determine the extent of the tract, associated ENT anomalies and for presurgical planning.

We present a case of CMCC in a neonate with description of clinical presentation and imaging features.

## 2. Case Presentation

A full term male neonate was born at 38 weeks of gestation with birth weight of 3465 grams. The infant's mother was a 20-year-old African American female with a history of obesity and pregnancy induced hypertension. Otherwise there was no history of tobacco, drug, or alcohol abuse and all the routine prenatal laboratory tests were normal.

**Figure 1:**
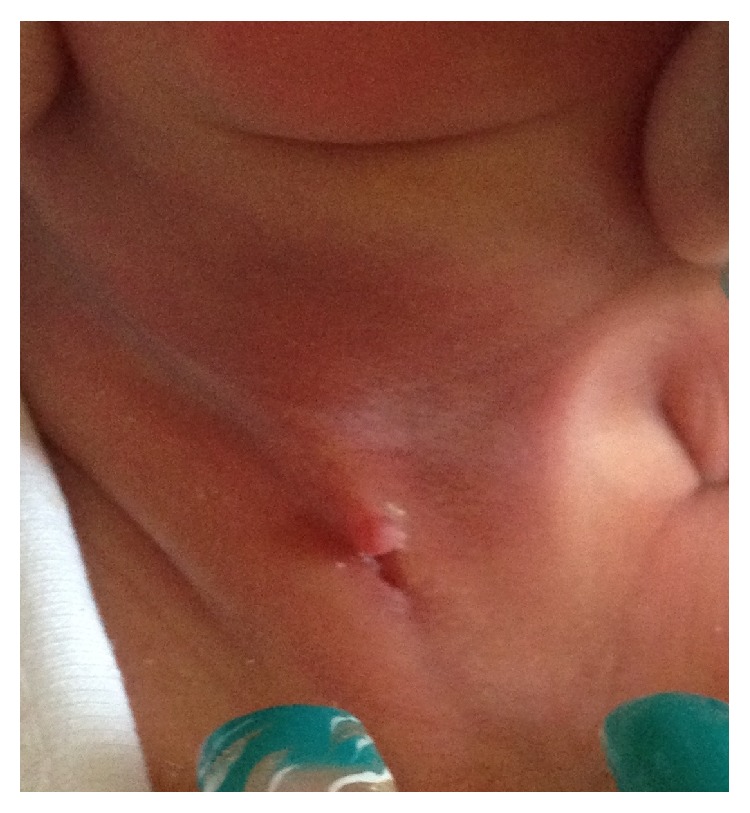
Linear craniocaudal pink track in the anterior neck with a skin tag arising from its superior aspect.

At birth, the child was examined and noted to have a linear craniocaudal pink track in the anterior neck with a skin tag arising from its superior aspect (Figure 1). No discharge was present from the track. A fibrous subcutaneous cord could be palpated beneath the track.

Because of the presence of a cervical cleft an MRI was requested to evaluate the cleft and to look for its internal extension and associated anomalies in the neck.

**Figure 2:**
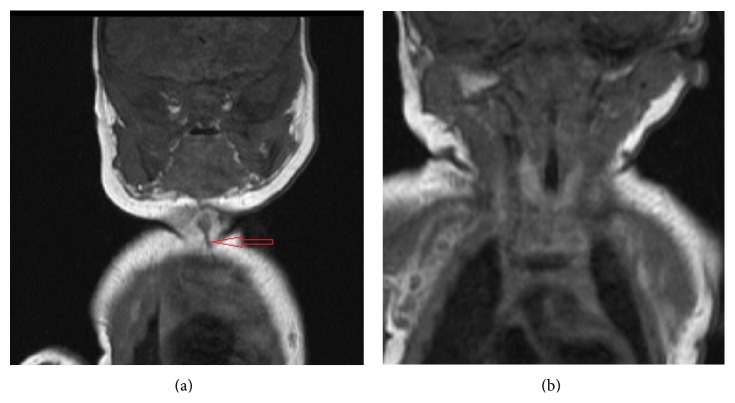
(a) Coronal T1 weighted MR image of neck demonstrates low T1 signal vertical midline cleft in cutaneous/subcutaneous region (open red arrow). (b) Coronal T1 image demonstrates normal right and left lobes of the thyroid.

**Figure 3:**
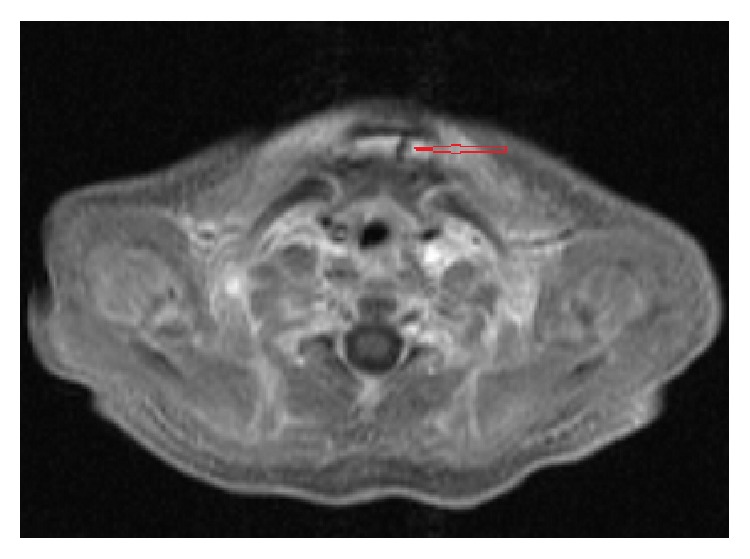
Postgadolinium fat saturated T1 axial MR image of the neck shows midline low T1 signal blind ending cleft in the skin/subcutaneous fat (red open arrow).

**Figure 4:**
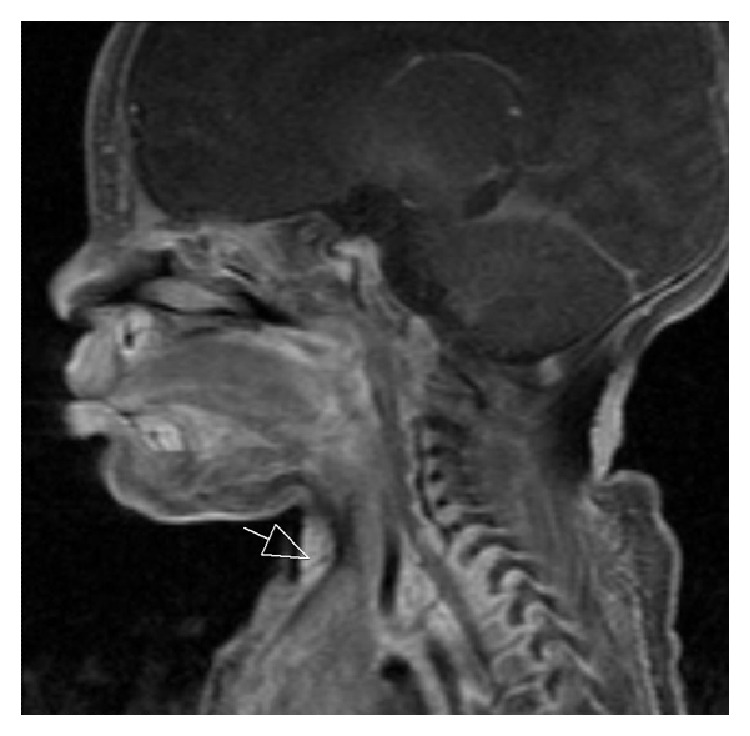
Sagittal T1, postgadolinium image shows small cervical tract (arrow) which terminates within the soft tissues and with no extension into deeper structures of the neck.

MRI with contrast was performed which demonstrated a defect in the cutaneous/subcutaneous soft tissues in the midline anterior neck, anterior to the strap muscles. The defect measured 2 cm in craniocaudal dimension and 0.5 cm in its width. It demonstrated low T1 and T2 signal and extended from the level of hyoid bone caudally to the level of manubrium (Figures 2, 3, and 4). No enhancement was seen in postgadolinium images. No extension to the sternum or connection with the deeper soft tissues was identified. The thyroid was normal in location, size, and enhancement. No cyst was identified in relation to thyroid or thyroglossal duct.

## 3. Discussion

Congenital midline cervical cleft (CMCC) is a rare congenital anomaly, which is thought to be due to failure of fusion of the first and second branchial arches during embryogenesis [4]. Less than 100 cases have been reported in the English literature. Most of the cases are reported to be sporadic in Caucasian females with female to male ratio of 2 : 1 [3]. The cleft is located in midline of the anterior neck anywhere between the mandible and the sternum. It may present as a midline defect of the anterior neck skin with a skin projection or sinus or as a subcutaneous fibrous cord. Although not a finding in our patient, at birth, the area often has a discharge [5] and is covered by thin often desquamating epithelium. Over the next few months the epithelium toughens and dries up. Histologically, the lesion consists of skeletal muscle, a fibrous cord, and exocrine tissues [6].

An incidence of 1-2% of congenital cervical malformations has been described in various case series; these include absence of thyroid, ectopic thyroid or thyroglossal duct cyst, ectopic bronchogenic cyst, branchial cyst, midline hemangioma, midline abdominal web, ectopia cordis, clefts of the lip, chin, tongue, mandible, or sternum, absence of hyoid bone or thyroid cartilage, and rarely congenital cardiac anomalies [6]. Our case had none of the other associations. Ultrasound is sometimes used as a first line imaging modality for other cervical anomalies. However, the full extent of the cervical tract and possible associated anomalies is best demonstrated with MRI. MRI is also useful for presurgical planning.

As the child grows or if there is inadequate repair, the fibrous cord becomes more conspicuous and can lead to contractures of the neck, torticollis, or limited extension [6]. Due to traction on the mandible, a bony prominence of the mandible can be palpated and exostosis of mandible or sternum can be seen on radiographs and/or MRI. Thus an early diagnosis is critical to avoid these complications.

Antibiotics may be required to treat infections or abscesses of congenital midline cervical clefts. Treatment is usually surgical excision and closure of the defect. Although exact time and technique of surgery are variable in different reports, most of the authors advocate earliest possible intervention for best results. Z-plasty incisions are recommended especially in cases of delayed presentation in order to prevent the development of scar and subsequent cicatrices contractures [6]. Primary anastomosis has been used with some success in patients with small clefts [7].

In summary, a congenital cervical cleft is a rare anomaly. A small number of these are associated with other midline anomalies of the head and neck and the chest. MRI is a useful modality for demonstrating the extent of the cleft, determining if there are any other associated anomalies, and it may be useful for presurgical planning for repair.
